# MyxoPortal: a database of myxobacterial genomic features

**DOI:** 10.1093/database/baae056

**Published:** 2024-07-02

**Authors:** Rayapadi G Swetha, Benita S Arakal, Santhosh Rajendran, K Sekar, David E Whitworth, Sudha Ramaiah, Philip E James, Paul G Livingstone, Anand Anbarasu

**Affiliations:** School of Bio-Sciences and Technology, Vellore Institute of Technology, Vellore Campus, Tiruvalam Road, Katpadi, Vellore, Tamil Nadu 632 014, India; School of Sports and Health Sciences, Cardiff Metropolitan University, Llandaff campus, Western Avenue, Cardiff CF5 2YB, UK; Department of Computational and Data Sciences, Indian Institute of Science, CV Raman Road, Bengaluru, Karnataka 560 012, India; Department of Computational and Data Sciences, Indian Institute of Science, CV Raman Road, Bengaluru, Karnataka 560 012, India; Department of Life Sciences, Aberystwyth University, Cledwyn Building, Penglais Campus, Aberystwyth, Ceredigion, Wales SY23 3FL, UK; School of Bio-Sciences and Technology, Vellore Institute of Technology, Vellore Campus, Tiruvalam Road, Katpadi, Vellore, Tamil Nadu 632 014, India; School of Sports and Health Sciences, Cardiff Metropolitan University, Llandaff campus, Western Avenue, Cardiff CF5 2YB, UK; School of Sports and Health Sciences, Cardiff Metropolitan University, Llandaff campus, Western Avenue, Cardiff CF5 2YB, UK; School of Bio-Sciences and Technology, Vellore Institute of Technology, Vellore Campus, Tiruvalam Road, Katpadi, Vellore, Tamil Nadu 632 014, India

## Abstract

Myxobacteria are predatory bacteria with antimicrobial activity, utilizing complex mechanisms to kill their prey and assimilate their macromolecules. Having large genomes encoding hundreds of secondary metabolites, hydrolytic enzymes and antimicrobial peptides, these organisms are widely studied for their antibiotic potential. MyxoPortal is a comprehensive genomic database hosting 262 genomes of myxobacterial strains. Datasets included provide genome annotations with gene locations, functions, amino acids and nucleotide sequences, allowing analysis of evolutionary and taxonomical relationships between strains and genes. Biosynthetic gene clusters are identified by AntiSMASH, and dbAMP-generated antimicrobial peptide sequences are included as a resource for novel antimicrobial discoveries, while curated datasets of CRISPR/Cas genes, regulatory protein sequences, and phage associated genes give useful insights into each strain’s biological properties. MyxoPortal is an intuitive open-source database that brings together application-oriented genomic features that can be used in taxonomy, evolution, predation and antimicrobial research. MyxoPortal can be accessed at http://dicsoft1.physics.iisc.ac.in/MyxoPortal/.

**Database URL:**  http://dicsoft1.physics.iisc.ac.in/MyxoPortal/.

**Graphical Abstract**

**Figure:**
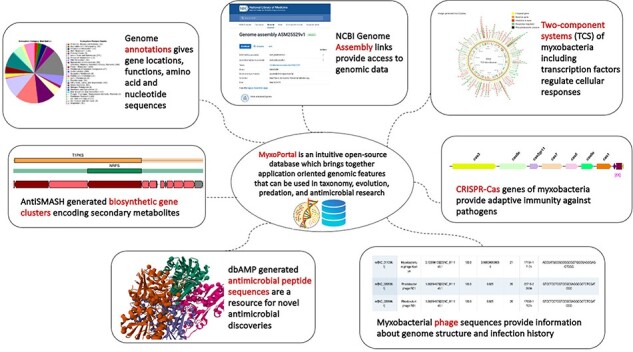

## Introduction

Myxobacteria are gram-negative ubiquitous predatory bacteria that are potent producers of secondary metabolites, lytic enzymes, outer membrane vesicles and antimicrobial peptides (1). These bacteria exhibit a social multicellular lifecycle employing two types of motilities—social (S-type) and adventurous (A-type) (2). They utilize a ‘wolf pack’ mechanism to kill their prey, wherein they secrete an antimicrobial arsenal that kills and digests prey cells with released macromolecules consumed thereafter (3, 4).

In 2006, the first myxobacterial genome sequence (*Myxococcus xanthus* DK 1622) was published, but as of late 2023, there are over 250 complete and draft myxobacterial genomes publicly available in the National Center for Biotechnology Information (NCBI) database (5). Genomes of myxobacteria are generally >10 Mb in size, with occasional exceptions like *Anaeromyxobacter spp*. (5 Mb) (6) and *Vulgatibacter incomptus* (4.4 Mb) (7). Downstream analyses of genomes have paved the way for functional genomics, comparative genomics and other post-genomics approaches, such as transcriptome and proteome analyses, for a better understanding of predatory mechanisms and their antimicrobial potential (8). Genome sequence analysis has also provided important insights into their taxonomy, including their recent reclassification as a novel phylum Myxococcota, with classes Myxococcia and Polyangia, orders Myxococcales, Haliangiales, Nannocystales and Polyangiales, families *Myxococcaceae, Vulgatibacteracea, Anaeromyxobacteracea, Polyangiaceae, Sandaracinaceae, Nannocystaceae* and *Haliangiaceae*, over 30 genera and 60 species (4).

Annotation of genomes gives the blueprint of an organism which then can be extrapolated to study their functional traits. Genomic annotations not only facilitate functional predictions but also enable investigations of strain-to-strain variations, and such studies have enlightened us regarding myxobacterial functional diversity (9). Genome-based studies have investigated the signalling pathways involved in carotenoid pigmentation, quorum signalling and fruiting body production (10). One-component system (OCS), two-component system (TCS), serine/threonine (Ser/Thr) kinases, transcriptional regulators (TRs), non-coding RNAs (ncRNAs) and alternative sigma factors have also been extensively studied (10–12), shedding light on the mechanisms involved in myxobacterial predation and cooperative behaviour. Although not widely studied in myxobacteria, bacteriophages (phages) have been isolated for *M. xanthus* (namely, MX-1, Mx4, Mx8 and Mx9) (13). Studies have shown the presence of phage protein-encoding genes in the genomes of myxobacteria (14) and tools like PHASTER, which identify phage genes in bacterial genomes, have greatly enhanced our understanding of myxobacterial phage (15). *Myxococcus xanthus* genomes also possess clustered regularly interspaced short palindromic repeat-associated (CRISPR-Cas) systems, which are involved in multicellular development and phage immunity (16).

There are more than 100 core structures and over 500 derivatives of secondary metabolites characterized in myxobacteria (17). While hundreds of these myxobacterial secondary metabolites have been explored via the traditional ‘grind and find’ strategy, genome sequence analysis, and genome mining have shown that there is huge untapped antimicrobial potential in these gifted microbes (18). This has changed research efforts to more of a ‘gene to compound’ strategy in the recent past (19). Several pieces of software, such as antibiotics & Secondary Metabolite Analysis SHell (antiSMASH) (20), Secondary Metabolite Unknown Regions Finder (SMURF) (21), CLUster SEquence ANalyzer (CLUSEAN) (22), ClustScan (23), Structure-Based Sequence Analysis of Polyketide Synthases (SBSPKS) (24), NRPSPredictor (25), and Natural Product searcher (NP searcher) (26) have been used to search for secondary metabolite gene clusters (27). Myxobacterial genome mining has shown the presence of numerous and diverse biosynthetic gene clusters (BGCs) that include non-ribosomal peptide synthetases (NRPS), polyketide synthases (PKS), terpenes and other metabolites with biosynthetic potential (28, 29). Furthermore, a similar approach has also led to the investigation of antimicrobial peptides (AMPs) that are increasingly studied to combat the global issue of antimicrobial resistance. AMPs eliminate pathogenic bacteria extracellularly by disrupting bacterial membranes or intracellularly through cross-talk with cellular components. Antimicrobial peptides have been sourced from bacteria, plants, invertebrates, amphibians and higher animals (30). Recent *in silico* studies have investigated the presence of promising AMPs from myxobacterial genomes (31).

Using genomic analyses to inspire experimental investigations can not only be a highly productive strategy for antimicrobial discovery but can also be very time-consuming for non-bioinformaticians. We therefore wanted to develop a resource hub for myxobacteria which would bring together, and provide easy access to, genomic sequence data and the results of diverse genetic analyses.

**Figure 1.**
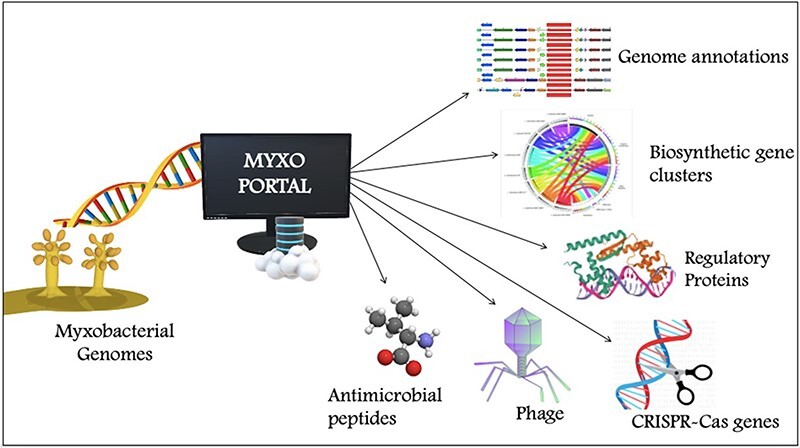
MyxoPortal work-flow diagram: the MyxoPortal database offers vast amounts of integrated genomic information, such as genome annotations, BGCs, AMPs, regulatory proteins (which include OCSs, TCSs, TFs and DNA-binding proteins), CRISPR genes and phage genes of myxobacteria.

To compile genomic datasets, including annotation of biosynthetic gene clusters, antimicrobial peptides, CRISPR genes, phage genes and regulatory proteins, a comprehensive database—MyxoPortal was developed (Figure 1). This database is aimed to expedite access to myxobacterial genome datasets by providing an all-in-one repository that can be easily accessed via a web browser (http://dicsoft1.physics.iisc.ac.in/MyxoPortal/).

## Methods

### Data collection

A total of 262 complete and draft genomes from the NCBI RefSeq database were downloaded for downstream analysis. Search terms included all known myxobacterial genera. Metagenome-assembled genomes (MAGs) were deliberately not included in the first version of MyxoPortal, as we wanted to initially focus on high-quality genomes from cultured strains of organisms which have been experimentally confirmed as being myxobacteria. Table 1 gives the list of genomes downloaded for analysis.

**Table 1.** List of myxobacterial genomes. Genomes (numbered within brackets) of fully characterized and uncharacterized species of myxobacteria included in the database

Genus	Fully characterized type species	Uncharacterized species
*Aggregicoccus*	–	1 (1)
*Anaeromyxobacter*	4 (26)	17 (26)
*Archangium*	3 (11)	5 (11)
*Byssovorax*	1 (2)	1 (2)
*Chondromyces*	2 (2)	–
*Corallococcus*	12 (59)	28 (59)
*Candidatus*	1 (4)	–
*Citreiococcus*	1 (1)	–
*Cystobacter*	3 (4)	–
*Enhygromyxa*	1 (3)	–
*Haliangium*	1 (4)	–
*Labilithrix*	1 (11)	–
*Melittangium*	1 (2)	–
*Minicystis*	1 (2)	1 (2)
*Myxococcus*	10 (66)	30 (66)
*Nannocystis*	2 (19)	6 (19)
*Pleiocystis*	1 (1)	1 (1)
*Polyangium*	4 (6)	1 (6)
*Pseudenhygromyxa*	–	1 (1)
*Pyxidicoccus*	3 (6)	–
*Sandaracinus*	1 (10)	8 (10)
*Sorangium*	1 (18)	2 (18)
*Stigmatella*	3 (5)	–
*Vitiosangium*	–	1 (1)
*Vulgatibacter*	1 (1)	–

### Genome annotations

Genomic sequences of all myxobacterial species were submitted in FASTA/GenBank file format to the RAST (Rapid Annotation using Subsystem Technology) web server (https://rast.nmpdr.org) (32–34). Once the genome was uploaded, contig statistics were computed by the system and additional taxonomic information, such as NCBI taxonomy ID, genus, species, etc. was inputted to verify the organism. The annotation pipeline used for annotating the genomes was RASTtk with default parameters and output spreadsheet files were downloaded in Excel (XLS) format.

### BGC prediction

Mining of BGCs was performed by uploading the genomic sequences (FASTA format) of all myxobacteria onto the antiSMASH web server (https://antismash.secondarymetabolites.org) version 7.0 (20). The detection strictness of well-defined clusters and partial clusters was set to relax, and extra features such as KnownClusterBlast, TFBS analysis, ActiveSiteFinder, SubClusterBlast and RREFinder features were enabled.

### Prokaryotic regulatory proteins

Regulatory proteins (RPs) such as transcription factors (TFs) and TCS are crucial biomolecules that have pivotal roles in prokaryotic organisms Using the P2RP (predicted prokaryotic regulatory proteins) webserver (http://www.p2rp.org) (35), genomic sequences of all myxobacterial species were queried against the regulatory proteins prediction tool for RP analysis. Distinct output files were generated for TFs, TCS and ODPs (other DNA-binding proteins) and downloaded in Excel (XLS) format.

### Bacterial phages

To detect and annotate prophage sequences within myxobacterial genomes, the PHASTER (PHAge Search Tool Enhanced Release) webserver (https://phaster.ca) was used (15). The PHASTER webserver tool was employed to predict mobile genetic elements such as prophages within bacterial genomes.

### CRISPR/Cas genes

Identification of CRISPR loci and Cas proteins within myxobacterial species was conducted using CRISPRminer version 1 (http://www.microbiome-bigdata.com/CRISPRminer/), which also features additional functional modules, such as novel class 2 CRISPR-Cas loci identification and anti-CRISPR protein prediction (36). FASTA genomic sequences for each myxobacterial genome were uploaded and detection was run using the CRISPRCasFinder algorithm.

### Antimicrobial peptides

AMPs are naturally occurring, host-defense peptides that can kill pathogenic microbes and are abundantly expressed from various animal and plant sources. To search for AMP sequences, myxobacterial genomes were uploaded to a data-mining server called dbAMP version 3.0 (https://awi.cuhk.edu.cn/dbAMP/index.php), and the AMPpredictor function was run (37).

### Database construction

The data obtained from the various genomics analyses described earlier were curated and compiled to create MyxoPortal. The data curation was done using Perl scripts, and MySQL was utilized to store and manage the processed data. Perl scripts used for the data curation are available in GitHub (https://github.com/mbclabvit/MyxoPortal). The graphical user interface for the MyxoPortal database was developed using HTML, JavaScript and CSS. The PERL/CGI and PERL/DBI modules were used to create the search engine. To achieve a faster response, the database was developed and hosted on a high-end server-class board and processor (Intel Xeon10 core Intel-based CPU E5-2630V4 @ 2.20 GHz) with CentOS7 operating platform (64 GB RAM). The database is powered by an Apache server. The database was thoroughly evaluated with a variety of browsers and operating systems, including Windows, Linux, and MAC. Users can access MyxoPortal via the most recent web browsers through the URL: http://dicsoft1.physics.iisc.ac.in/MyxoPortal/.

## Results and discussion

### MyxoPortal structure

**Figure 2.**
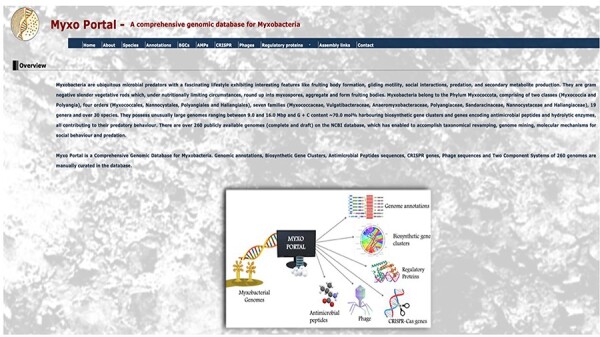
MyxoPortal homepage. The home section provides information on collaborators involved in the MyxoPortal database project. The About section displays an overview containing summarized information on myxobacteria including a work-flow diagram. The Species section indicates a table displaying the data files for all myxobacterial species available on the database. Certain sections have been condensed for display purposes.

MyxoPortal is a freely accessible, user-friendly, online database which does not require registering to use. This provides the user with easy, uninterrupted, and rapid access to data. The database has seven datasets (icons), a list of genera and species (species), annotations of all coding regions (nucleotide and amino acid sequences) for all myxobacterial genomes (annotations), plus predicted BGCs, AMPs, CRISPR-cas genes (CRISPR), phage-associated sequences (Phages) and RPs) for each genome. Clicking the ‘Enter’ icon takes the user to the database’s home page (Figure 2). The home page gives some basic information on myxobacteria. This page also has a banner containing various options with clickable icons, linking to the Home, About, Species, Annotations, BGCs, AMPs, CRISPR, Phages, RPs and Assembly datasets/pages mentioned earlier. The user can navigate through the desired sections and on selecting them, is then directed to a page containing the list of all 266 myxobacterial species, as shown in Table 1.

### MyxoPortal navigation

There are multiple ways to navigate the data within MyxoPortal. The user can select the ‘Species’ option, type the genus/species name and go straight to the desired genus or species of myxobacterium and look further into the desired datasets. This approach helps the user to screen the list to get to their genus/species/strain of interest avoiding searching individually. Also, the database allows the user to get the information of all the sequenced genomes within a genus or species which would be a time-consuming exercise otherwise. The user need not download the genomes to do downstream analysis as most analyses are readily available. However, if the user needs to download the genomes for any further analysis that is not covered within the database, this can be done easily by going to the assembly links. The links to the genomes on the home page will take the user to the NCBI database where the genomes can be acquired.

**Figure 3.**
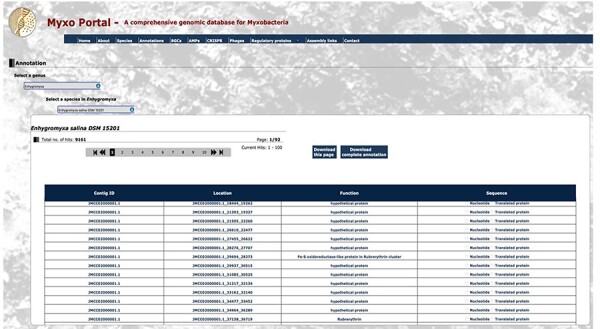
Snippet of the annotation output file for *Enhygromyxa salina* DSM 15201 from RAST. The annotation file for myxobacterium *Enhygromyxa salina* DSM 15201 displays 9161 hits with location, function, nucleotide sequence and protein sequence for each contig. Certain sections have been condensed for display purposes.

All myxobacterial genomes deposited in GenBank before October 2023 were used to create the MyxoPortal database. By selecting the genome of a strain of myxobacterium, the user is automatically directed to the respective output page that displays the required information in detail. On the output page of each search, the user can download the displayed results in comma-separated values format. For example, if the user wishes to obtain genome annotations for *Enhygromyxa salina* DSM 15201, the user would select the option ‘Genome Annotation files’, which would direct them to the myxobacteria list, within which they select the strain ‘*Enhygromyxa salina* DSM 15201’. This automatically loads up the page containing the RAST annotation table for *Enhygromyxa salina* DSM 15201, which details the contig ID, function, nucleotide and amino acid sequences for each of the genes present within the genome (Figure 3). Alternatively, in the ‘Species’ tab, a list of the genera is generated which further aids in choosing the desired species/strain. Also, the total count of data files for each category, such as Genome Annotations, BGCs, AMPs, Phages and RPs, is displayed for each species/strain. By clicking the total number, the interface will display the respective data.

### MyxoPortal provides rapid access to a rich compendium of genomic information

Diverse pieces of software and databases, such as RAST, antiSMASH, P2RP, PHASTER, CRISPRminer, dbAMP and AMPpredictor were used to generate the datasets within MyxoPortal, using all myxobacterial genome sequences available at the time of this study. Bringing together such datasets will facilitate their use for further analysis. For example, the user may wish to compare species of myxobacteria from marine and terrestrial sources, looking at the secondary metabolite datasets. The BGC data generated by antiSMASH is useful for comparing the BGCs from diverse sources with their myxobacterial counterparts, and invaluable in antimicrobial discovery studies. Although BGC datasets can easily be derived by using the antiSMASH server, by compiling all the information on the BGCs of myxobacteria in one place, downloadable in simple excel formats, MyxoPortal facilitates comparative studies of BGC data. MyxoPortal datasets also provide nucleotide and amino acid sequences for proteins and metabolites, which can be used for phylogenomic studies. For instance, the phage protein sequences enable users to investigate evolutionary aspects of myxobacterial biology. Importantly, MyxoPortal datasets have a download function, allowing users to work with their selected proteins/genes offline. We will continue to update MyxoPortal as further myxobacterial genomes are submitted to Genbank and are working on allowing the database to update itself automatically.

### MyxoPortal—a unique database

Generally, all software and databases are subject to maintenance that involves expertise and finance. This has in turn resulted in some software/databases being redundant. In 2007, Arshinoff *et al*. designed a model organism database called Xanthusbase that consisted of genomic data and annotations for *M. xanthus* (38). In addition to Xanthusbase, they implemented another database called MyxoPedia, which utilized a Wikipedia approach that provided scientific literature dedicated to *M. xanthus*. Both these databases are currently inaccessible. MyxoDB, a recently developed database has datasets on natural products derived from myxobacterial species, listing the bioactivity, structure and physical and chemical properties of the secondary metabolites (39). MyxoDB with datasets on NMR-based metabolomics not only helped to identify secondary metabolites with potent antimicrobial activities but also facilitated the identification of novel compounds. Analogously, MyxoPortal is designed to assist the ‘Gene to Compound’ approach, which uses high-throughput genome mining to facilitate the discovery of novel antimicrobial compounds.

### Applications of MyxoPortal

MyxoPortal is a resource hub for application-oriented studies. Myxobacteria have been extensively studied to understand their multicellular life cycle and predatory mechanisms (40), as well as screening them for secondary metabolites, enzymes and antimicrobial peptides (31, 41). With the ongoing discovery of novel myxobacterial phyla, comparative analysis of genome sequences becomes an increasingly laborious task. Moreover, such analysis requires computational skills that biologists may find overwhelming and may hamper the quality of research. MyxoPortal is a useful resource for such comparisons, as it brings together the results of diverse analyses in one place, enabling further analyses and inspiring wet laboratory experiments. The database will serve as a tool for high-throughput analysis to study the biological functions of myxobacteria and their antimicrobial potential, which is of paramount importance in the era of antimicrobial resistance. Studies using high-throughput genomic analyses to direct application-oriented experimental studies in this way, have proven to be extremely powerful in recent times (19, 20, 31).

Future updates to MyxoPortal will incorporate data for the genomes of newly sequenced myxobacterial strains and possibly MAGs, plus extend analyses to include further genomic features such as plasmids and transposons. If users desire the inclusion of additional features in future versions of MyxoPortal (or wish to report bugs/broken links), they are encouraged to contact the authors. Alternatively, a bug/feature report option is provided in the contact page for users to report any issues.

## Conclusion

MyxoPortal includes all myxobacterial genomes which were publicly available as of October 2023. It is an intuitive open-source database that brings together application-oriented genomic features that can be used in taxonomy, evolution, predation and antimicrobial research, and has been designed to facilitate the discovery of novel antimicrobials.

## Data Availability

The data underlying this article can be accessed at http://dicsoft1.physics.iisc.ac.in/MyxoPortal/
